# 

**Published:** 1896-01

**Authors:** 


### BOOKS RECEIVED.

Injuries and Diseases of the Genital and Urinary Organs. By Henry Morris, M. A., M. B., Lond., F. R. C. S., Surgeon to, and Lecturer on, Surgery at the Middlesex Hospital ; Member of the Council and of the Court of Examiners of the Royal College of Surgeons, England, etc. Small 8vo, pp. xvi.—478. With ninety-seven illustrations. New York : William Wood & Co. 1895.

Outlines of Materia Medica and Pharmacology. A Text-book for Students. By H. M. Bracken, M. D., Professor of Materia Medica, Therapeutics and Clinical Medicine, University of Minnesota, Octavo, pp. 383. Price, $2.75. Philadelphia : P. Blakiston, Son & Co., 1012 Walnut street. 1895.

Hand-Book for Hospitals. By Abby Howland Woolsey, Member of the Committee on Hospitals, State Charities Aid Association. No. 32 New York State Charities Aid Association Series. Third edition. Price, $1.00. G. P. Putnam's Sons, New York, 27 West Twenty-third street. 1895.

Transactions of the Texas State Medical Association. Twenty-seventh annual session, held at Dallas, Texas, April 23, 24, 25, 26, 1895. H. A. West, M. D., secretary, Galveston, Texas. Knapp Bros., Printers and Publishers. 1895.

A System of Surgery. By American Authors. Edited by Frederic S. Dennis, M. D., Professor of the Principles and Practice of Surgery, Bellevue Hospital Medical College, New York ; President of the American Surgical Association, etc.; assisted by John S. Billings, M. D., LL. D., D. C. L., Deputy Surgeon-General, U. S. A. To be completed in four imperial octavo volumes, containing about 900 pages each, with index. Profusely illustrated with figures in colors and in black. Volume III., 908 pages, 207 engravings and ten colored plates. Price, per volume, $6 in cloth ; $7 in leather ; $8.50 in half morocco, gilt back and top. For sale by subscription. Full circular free to any address on application to the publishers, Lea Brothers & Co., Philadelphia.

Surgery. A Practical Treatise, with Special Reference to Treatment. By C. W. Mansell Moullin, M. A., M. D., Oxon. ; Fellow of the Royal College of Surgeons ; Surgeon and Lecturer on Physiology to the London Hospital, etc., assisted by various writers on special subjects. With 623 illustrations. Royal 8vo, pp. 1250. Third American edition, revised and edited by John B. Hamilton, M. D., LL. D., Professor of the Principles of Surgery and Clinical Surgery, Rush Medical College, Chicago ; Professor of Surgery, Chicago Polyclinic ; Surgeon, formerly Supervising Surgeon-General, U. S. Marine Hospital Service, etc., etc. Price, $6.00, Philadelphia: P. Blakiston, Son & Co., 1012 Walnut street. 1895.

An Atlas of Ophthalmoscopy. With an Introduction to the Use of the Ophthalmoscope. By Dr. O. Haab, Professor of Ophthalmology, University of Zurich. Translated and edited by Ernest Clark, M. D., B. S. (Lond.) ; Fellow of the Royal College of Surgeons ; Surgeon to the Central London Ophthalmic Hospital, etc. Duodecimo, profusely illustrated. New York : William Wood & Company. 1895.

A Guide to the Practical Examination of Urine, for the Use of Physicians and Students. By James Tyson, M. D., Professor of Clinical Medicine in the University of Pennsylvania, and Physician to the Hospital of the University, etc. Duodecimo, pp. xii.—276. Ninth edition, revised and corrected. With a colored plate and wood engravings. Price, $1.25. Philadelphia: P. Blakiston, Son & Co., 1012 Walnut street. 1895.

A Hand-Book of Obstetric Nursing. For Nurses, Students and Mothers. Comprising the course of instruction in obstetric nursing given to the pupils of the Training School for Nurses connected with the Woman's Hospital of Philadelphia. By Anna M. Fullerton, M. D., Physician in charge of, and Obstetrician, Gynecologist and Surgeon to the Woman's Hospital of Philadelphia, etc. Fourth, revised edition. Illustrated. Price, $1.00. Philadelphia : P. Blakiston, Son & Co., 1012 Walnut street. 1895.

Spectacles and Eyeglasses. Their Forms, Mounting and Proper Adjustment. By R. J. Phillips, M. D., Adjunct Professor of Diseases of the Eye, Philadelphia Polyclinic and College for Graduates in Medicine ; Ophthalmic Surgeon to the Presbyterian Hospital in Philadelphia, etc. Second edition, revised, with forty-nine illustrations. Price, $1.00. Philadelphia : P. Blakiston, Son & Co., 1012 Walnut street. 1895.

Obstetrical Pocket Phantom. By Dr. K. Shibata, Specialist in Gynecology and Obstetrics, Tokio, Japan ; Physician to the Woman's Clinic at the University of Munich. Preface by Prof. Franz von Winckel. With eight illustrations, one pelvis and two-jointed manikins. Translated from the third edition by Ada Howard-Andenried, M. D., Physician to the Children's Clinic at the Woman's Hospital, Philadelphia. Price, $1.00. Philadelphia: P. Blakiston, Son & Co., 1012 Walnut street. 1895.

Proceedings of the Orleans Parish Medical Society for 1894. Augustus McShane, M. D., Secretary. Volume II. New Orleans : L. Graham & Son, Printers. 1895.

The Johns Hopkins Hospital Reports. Volume IV., number 9. Report in Pathology, IV. Contents, Deciduoma Malignum. By J. Whitridge Williams, M. D. Baltimore : The Johns Hopkins Press. 1895.

A Manual of the Practice of Medicine. By George Roe Lockwood, M. D., Professor of Practice in the Woman's Medical College of the New York Infirmary ; Attending Physician to the Colored Hospital and to the City (late Charity) Hospital, etc., etc. Small 8vo, pp. 935. With seventy-five illustrations in the text and twenty-two full-page colored plates. Price, $2.50. Philadelphia : W. B. Saunders, 925 Walnut street. 1895.

Principles of Surgery. By N. Senn, M. D., Ph. D., LL. D., Professor of Practice of Surgery and Clinical Surgery in Rush Medical College, Chicago ; Professor of Surgery in the Chicago Polyclinic ; Attending Surgeon to the Presbyterian Hospital ; Surgeon-in-Chief to St. Joseph's Hospital ; Ex-President American Surgical Association, etc., etc. Second edltion, thoroughly revised. Illustrated with 178 wood-engravings and five colored plates. Royal octavo, pp. xvi.—656. Extra cloth, $4.50 net ; sheep or half-Russia, $5.50 net. Philadelphia : The F. A. Davis Co., Publishers, 1914 and 1916 Cherry street.

